# Phlegmasia Cerulea Dolens in a Patient with Breast Cancer and Inferior Vena Cava Hypoplasia

**DOI:** 10.1155/2020/2176848

**Published:** 2020-02-27

**Authors:** A. Bianchi, S. Pozza, L. Giovannacci, Jos. C. van den Berg

**Affiliations:** ^1^Department of Visceral and Vascular Surgery, Ospedale Regionale di Lugano-CH, Switzerland; ^2^Department of Interventional Radiology, Ospedale Regionale di Lugano-CH, Switzerland

## Abstract

**Background:**

No clear guidelines exist for the management of phlegmasia cerulea dolens. This case report shows how a hybrid approach might be successful. It also shows how rare pathologies can combine to create a life- and limb-threatening condition. *Case Presentation*. A 75-year-old man, known for nephrotic syndrome currently under investigation, presented to the emergency department with a 24-hour history of left leg swelling followed by intense pain. The left lower limb showed a phlegmasia cerulean dolens. Renal function, coagulation profile, and inflammatory parameters were normal; D-Dimers 5,6 mg/L. The CT scan showed juxtarenal thrombosis of the hypoplastic IVC, involving both renal veins, reaching the left iliac-femoral-popliteal axis, with collateralization to the pelvic and mesenteric veins, associated with bilateral segmental pulmonary embolisms. A suspected left breast nodule was also found. Intravenous heparin was immediately administered, and urgent hybrid procedure with surgical thrombectomy and venous angiography and thromboaspiration, liberating the iliolumbar collaterals, was performed. A lateral leg fasciotomy was mandatory due to the phlegmasia cerulea. Postoperative Doppler US showed a good venous compressibility of the left leg. Thrombophilia screening was negative. The breast nodule was biopsied showing an invasive ductal carcinoma. The patient was discharged with oral rivaroxaban and indication for left mastectomy and oncological therapy with aromatase inhibitors.

**Conclusion:**

This case highlights the dramatic consequence of different risk factors for venous thromboembolism as cancer and nephrotic syndrome in a patient with hypoplasia of the inferior cava vein. Venous thromboaspiration has been used in order to timely recanalize important collaterals. Phlegmasia cerulea dolens was resolved after the procedure and lateral calf fasciotomy. Further evidence is needed to clearly define the role of venous thromboaspiration in the treatment of complex proximal deep venous thrombosis of the lower extremity.

## 1. Case Presentation

We present the case of a 75-year-old healthy male patient, who arrived at our Emergency Department for excruciating pain and swelling of the left leg that, when measured, resulted 1,5 times bigger than the right one.

The pain started the day before and worsened during the day, as the diameter of the leg was also increasing. He did not report any previous trauma. The only relevant anamnestic data was a short flight 4 weeks before.

The patient's past medical history was characterized by type II diabetes, hypertension, COPD, and also a nephrotic syndrome, recently diagnosed and still under investigation. His cardiovascular risk factors were cigarette smoking and arterial hypertension.

**Figure 1:**
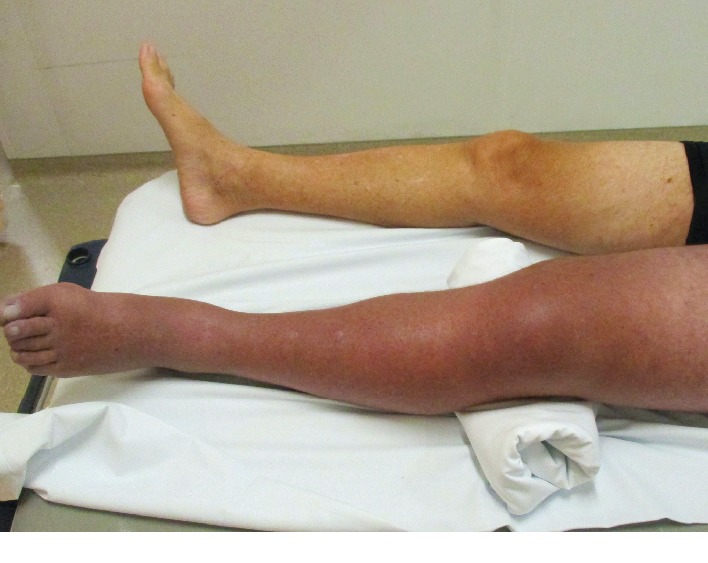
Picture showing the difference between the right normal leg and the left leg with a classic pattern of phlegmasia cerulea dolens.

At the clinical examination, the left leg presented typical signs of phlegmasia cerulea dolens (Figure 1): distal pulses were not palpable, and the calf was swollen, reddened, and painful, typical for a compartment syndrome.

**Figure 2:**
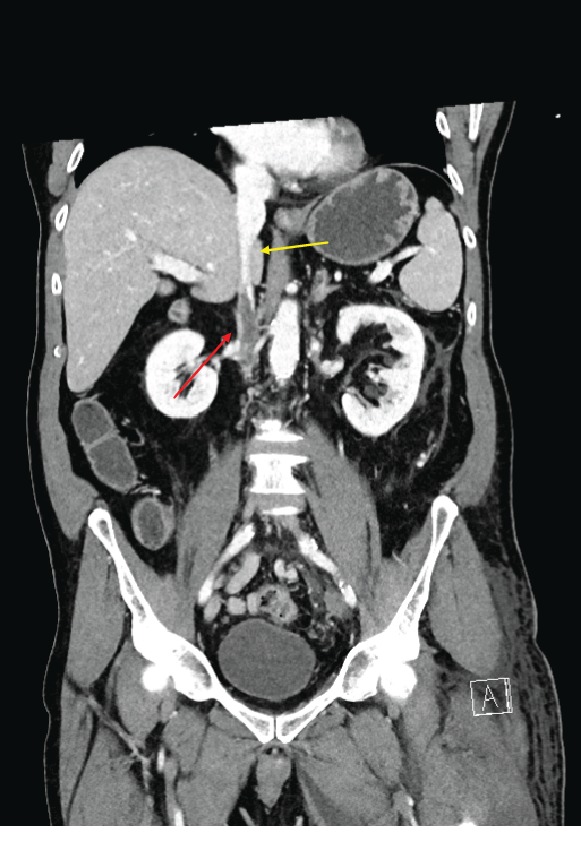
Angio-CT showing inferior vein cava thrombosis (red arrow) and inferior vein cava hypoplasia (yellow arrow).

**Figure 3:**
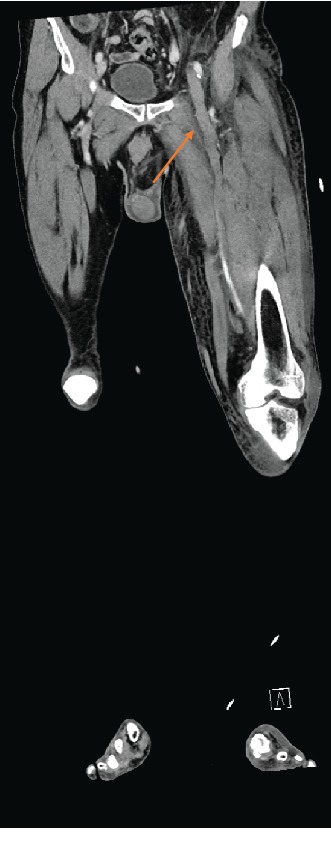
Angio-CT of the patient showing complete thrombosis of the left femoral vein (arrow).

An urgent angio-CT scan was performed and showed an extensive thrombosis of both renal veins, a hypoplasia of the inferior vena cava (Figure 2) extending from the renal veins to the diaphragm, and a complete thrombosis of the deep venous system of the left leg (Figure 3).

The venous drainage was partially granted by collaterals, the major being a lumbar vein, which drained into the superior mesenteric vein.

The CT scan also showed a minimal bilateral basilar pulmonary embolism, a mass in the left mammary gland, and also an exceptionally big left inguinal hernia.

We decided to perform an iliofemoral venous thrombectomy associated with an intraoperative phlebography and venous PTA. The phlebography showed thrombotic material in the external iliac vein. The common iliac vein was occluded, determining a retrograde flux in the femoral vein. A thrombectomy with Fogarty catheter was performed, followed by a percutaneous angioplasty with the restoration of an anterograde flux. Due to the presence of good collateral circles, it was not necessary to liberate the renal veins. Because of the presence of a compartment syndrome, a fasciotomy of anterior and lateral compartments was also performed.

Postoperatively, the patient was transferred to the Intensive Care Unit and the clinical evolution was favorable.

**Figure 4:**
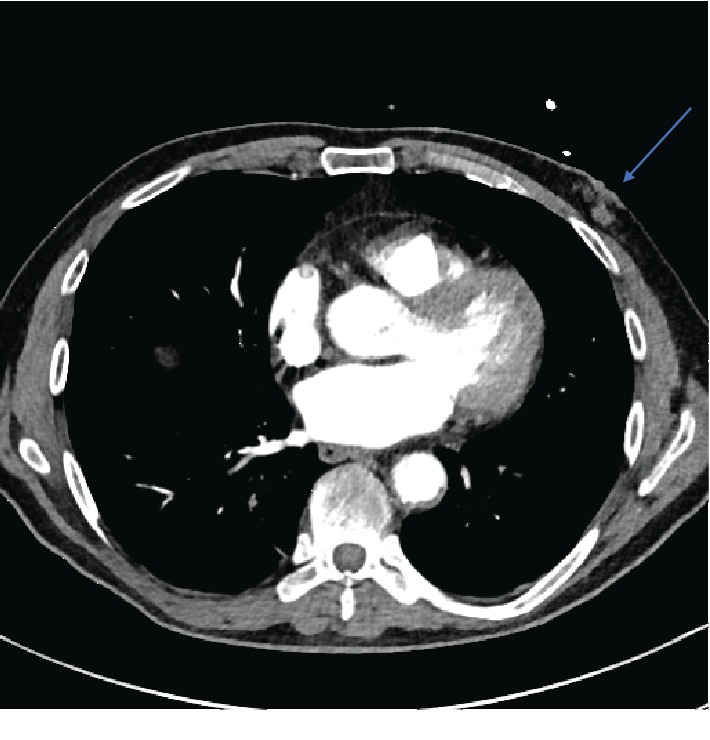
Angio-CT showing a left mammary mass (blue arrow).

At this point, the cause of this massive venous thrombosis had to be found, also considering the presence of a probable breast cancer (Figure 4).

The thrombophilia screening did not show any pathologic finding. The mammary lesion was on the inferior external quadrant of the left breast with retraction of the superimposed skin. After discussion with the OB/GYN specialist, a tru-cut biopsy was performed. The histopathological examination showed an invasive ductal carcinoma B.R.E. 2 with expression of estrogenic and progestin receptors at 90%, Ki-67 10%, C-erb-2 score 2+. Radical mastectomy was highly recommended, but the patient went back to his original country and we do not know if the operation was actually performed.

The hospital stay was regular, without any complication. A life-long oral anticoagulation with rivaroxaban 15 mg 1/day was started.

## 2. Discussion

This case gives us the chance to focus on three rare pathologies: phlegmasia cerulea dolens, inferior vena cava hypoplasia, and breast cancer in male patients.

Phlegmasia cerulean dolens is a rare condition, whose prevalence is unknown, and it represents the intermediate state between venous thrombosis and phlegmasia alba dolens. This condition was first described by Hildanus et al. in 1593 [1]. Etiopathogenesis was clarified in the first years of XX century by Crueveilhier [2] and Buerger [3]. The classical triad of symptoms, pain, swelling, and discoloration, was first described by Gregoire [4] in 1938. It is a severe condition which, even nowadays, is burdened by an amputation rate of 20-50% [5–7].

The literature about this condition is poor, and guidelines do not exist [8].

The prevalence is higher in men than in women, with a 1,5 : 1 ratio [9]. In 33% of cases [8], as in our case, a neoplastic disorder is found, which determines a paraneoplastic syndrome. In 50% of cases, a thrombophilic status, venous stasis, or oral contraceptives are found or used. In 16% of cases, the etiology is not found. Furthermore, for unknown reasons, the left leg is of interest in 45% of cases, while the right one only in 29%. Bilateral disease is found in 26%. One-third of the patients have a pulmonary embolism at admission.

Diagnosis is mainly clinical and the previously reported classic triad is preset. The radiological imaging of choice is a color Doppler ultrasound, eventually associated with angio-CT scan. Ultrasound is the exam of choice because it is widely available, not invasive, and without radiations. On the other hand, it is operator-related and is also difficult to find pelvic masses causing compression of venous axes.

Phlegmasia may be classified as not complicated, with incipient venous gangrene and with established venous gangrene. Morbidity and mortality increase as the clinical stage gets worse.

As already stated, no guidelines are available. As a consequence, the therapeutic choices are addressed to thrombus reduction and limb salvage. Two other main goals are the prevention of thrombus propagation and the maintenance of the venous axis patency.

Initial treatment is based on general support treatment and heparin infusion [9] in therapeutic range, which means that the aPTT has to be two times higher than normal reference values. Platelet count is mandatory to surveil heparin-induced thrombocytopenia (HIT syndrome).

If no clinical improvement occurs within few hours, normally 12 [9], a more aggressive treatment is needed. In this kind of pathology, as in the rest of the vascular surgery, there is a clear augmentation of endovascular procedures, both lysis or stenting, to the detriment of open venous thrombectomy. Open approach has the advantage to be a fast resolution of venous hypertension and also reduces thrombus propagation. However, this is a major invasive procedure, which needs general anesthesia and it is at high risk of rethrombosis. Endovascular approach, both lysis or mechanical thrombectomy, is less invasive and may grant a major permeability of micro circulation. The major disadvantage is the risk of pulmonary embolism due to clot fragmentation.

Vena cava anomalies have an incidence of 0,05-8,7% [10] in Caucasian population, sometimes associated with other vascular anomalies. Agenesis or hypoplasia of the inferior vena cava has a prevalence of 0,0005%. This condition is a risk factor for deep venous thrombosis: in fact, deep venous thrombosis in patient younger than 30 is 5%, largely superior to 0,5% of the health population of the same age. This difference is attributable to rheological phenomena, as stated by Virchow's triad.

Breast cancer in men has an incidence of 0,5-1% in Caucasian population [11], different from some areas of Central Africa where it shows an incidence up to 6% [11].

Hormonal imbalance between estrogens and androgens may play a major role in this rare condition. Hormonal alterations may be caused by a genetic condition, as in Klinefelter syndrome, or by acquired conditions, as in hepatic diseases, thyroid dysfunction, and obesity. A relationship exists between testicular pathology and mammary neoplasia: the most accredited hypothesis in literature is that diseases such as orchitis, retained testicles, and testicular lesions cause a reduction in androgen production and a consequent increase of estrogen production.

Breast cancer is associated with 9-14% increase of thrombophilia due to an increase in endogenous carbon monoxide production, known to enhance plasmatic coagulation both in vivo and in vitro, determining a higher risk for thrombosis in affected patients [12].

The severe condition presented by the patient is due to the combination of two strong risk factors for venous thrombosis, such as hypoplastic vena cava and breast cancer, that determined a rare clinical presentation.

## 3. Conclusion

To summarize, the treatment of phlegmasia cerulea dolens is still not codified by precise protocols. A recent review points out some milestones:

1. Early anticoagulation
2. Unclogging, if possible, the major venous axes
3. Active research of thrombophilia causes, especially neoplasia, and treatment

In the reported case, our treatment was coherent with those principles, aimed at avoiding limb amputation, which is still the major complication of phlegmasia, with an estimate rate of 20%.

In the future, especially if we analyze actual trend in vascular surgery, we shall see a major use of endovascular procedures, thanks to new drugs and new devices, at the expense of the traditional open techniques. Nowadays, clear evidences in the literature are yet to be found.
